# A bibliometric analysis and knowledge mapping of cognitive behavioral therapy research in depression over 20 years

**DOI:** 10.1097/MD.0000000000048780

**Published:** 2026-05-29

**Authors:** Shaokui Kan, Xinying Li, Nuannuan Chen, Yingli Zhang

**Affiliations:** a Guangzhou Civil Affairs Bureau Psychiatric Hospital/ Guangzhou Kangning Hospital, Guangzhou, China; b Depressive Disorders Ward, Shenzhen Kangning Hospital/Shenzhen Mental Health Center/Shenzhen Clinical Research Center for Mental Disorders, Shenzhen, China.

**Keywords:** bibliometric, CiteSpace, cognitive behavioral therapy, depression, visual analysis, VOSviewer

## Abstract

**Background:**

Depression negatively affects well-being, leading to lower life satisfaction, impaired social and psychological functioning, and greater disability. Cognitive behavioral therapy has been proven effective across various levels and populations of depression. This study employs bibliometric analysis to clarify research trends, summarize historical and current directions, and predict future trends, aiding researchers and policymakers in effectively prioritizing research resources.

**Methods:**

To identify publications related to depression and cognitive behavioral therapy (CBT), we performed a computerized search of the Web of Science Core Collection. Bibliometric analyses were conducted on multiple dimensions – including authors, journals, institutions, countries/regions, keywords, and references – using Excel 365, CiteSpace, Pajek, and VOSviewer.

**Results:**

Between 2005 and 2024, 6384 publications were analyzed. Publication trends showed a gradual rise, peaking at 494 publications in 2020, and subsequently experiencing a slow decline. Citations in this field demonstrated annual growth, reaching a peak of 33,882 citations in 2021, followed by a subsequent decline. The United States and the United Kingdom emerged as the leading countries in this research area, ranking highest in both publication output and citation counts. They host institutions that contribute significantly to publications and citations. Notable authors in this area include Gerhard Andersson, Pim Cuijpers, Per Carlbring, and Steven D. Hollon. Key journals publishing research in this area include the Journal of Affective Disorders, Behavior Research and Therapy, Mindfulness, Journal of Consulting and Clinical Psychology, and Psychological Medicine. Common keywords within this field are depression, followed by cognitive therapy, anxiety, psychotherapy, cognitive-behavior therapy, meta-analysis, mindfulness, randomized controlled-trial, symptoms, major depression, cognitive-behavioral therapy, efficacy, validation, cognitive behavior therapy, prevention, disorders, prevalence, psychometric properties, quality-of-life, and scale. These keywords indicate a sustained interest among researchers in cognitive interventions, treatment efficacy, preventive measures, and quality of life assessments. Furthermore, these research hotspots indicate that the psychometric properties and validation of treatments for depression are significant directions for future studies.

**Conclusions:**

This study employs bibliometric analysis to map research trends in CBT for depression, highlighting emerging trends and providing insights for researchers. It underscores CBT’s efficacy, adaptations, and integration with other therapies, emphasizing the need for continued research and interdisciplinary collaboration.

## 1. Introduction

Depression is characterized by persistent low mood and diminished interest in activities, severely affecting interpersonal relationships, academic achievement, and occupational functioning. Approximately 3.8% of the world’s population experiences depression, with prevalence among adults estimated at 5% (4% in men and 6% in women).^[1]^ In individuals aged 60 years and older, the prevalence increases to 5.7%.^[1]^ Additionally, 34% of adolescents aged 10 to 19 are at risk of developing clinical depression, with the highest prevalence rates found in the Middle East, Africa, and Asia.^[2]^ Notably, the incidence of depression in women is about 50% higher than in men. Worldwide, more than 10% of women experience depression during pregnancy or in the postpartum period.^[3]^

Depressive disorder is linked to reduced well-being, impaired social and psychological functioning, and higher disability levels, and is closely associated with suicide.^[4]^ Moreover, depressive disorders impose substantial societal burdens and rank among the foremost causes of disability globally.^[5]^ Presently, depression treatment approaches predominantly encompass pharmacotherapy, psychotherapy, and physical therapy. Psychotherapy assumes a pivotal role, particularly in addressing depression characterized by specific psychosocial stressors and cognitive deficits.^[6]^ In both the 2022 guidelines^[7]^ from the National Institute for Health and Care Excellence and the 2023 update^[8]^ from the Canadian Network for Mood and Anxiety Treatments, psychotherapy is recommended as a treatment for depression. Specifically, for mild to moderate depression, psychotherapy is the preferred initial treatment approach. Recommended interventions comprise cognitive behavioral therapy (CBT), interpersonal psychotherapy (IPT), guided self-help programs, and group-based therapy. CBT integrates both behavior therapy (BT) and cognitive therapy (CT) into a cohesive series of therapeutic approaches.^[9,10]^ At its core, CBT posits that various dysfunctional or distorted thought patterns contribute to or worsen maladaptive emotions and behaviors. Thus, CBT aims to pinpoint and address specific distorted automatic thoughts, maladaptive assumptions, and negative or dysfunctional patterns, ultimately reconstructing a more pragmatic and rational cognitive framework. By fostering the development of new coping strategies, CBT effectively and enduringly alleviates psychological distress. Historical research^[11–14]^ has repeatedly affirmed the efficacy of CBT in the treatment of depressive disorders. In a systematic review and network meta-analysis conducted by José A et al^[15]^ in 2018, they included 91 studies and found that CBT interventions significantly reduced depression scale scores more than conventional treatments in the short term. Additionally, research found that different forms of CBT, such as multimedia and hybrid formats, were effective in treating depression.^[12,16,17]^ In the study conducted by Matthijs O and colleagues^[18]^ in 2019, a meta-regression analysis examined the effectiveness of CBT in treating depression among children and adolescents. The analysis included 31 studies with a total of 4335 participants. Results indicated that CBT significantly reduced the severity of depressive symptoms and decreased the risk of depression by 63% during follow-up. Moreover, interventions incorporating behavioral activation and cognitive challenging techniques showed enhanced efficacy. Importantly, CBT also demonstrated a reduction in suicidal risk among adolescents.^[19]^ Furthermore, recent multicenter randomized double-blind controlled trial,^[20]^ along with systematic reviews^[21]^ employing network meta-analysis, have consistently affirmed the efficacy of CBT in addressing depression among older adults. Moreover, CBT has demonstrated effectiveness as a therapeutic approach for depression occurring during critical phases in women’s lives, such as pregnancy and the postpartum period.^[22–24]^

Bibliometric research utilizes both qualitative and quantitative approaches to examine, evaluate, and manage scholarly literature and information sources.^[25]^ Drawing upon the fields of information science, statistics, and computer science, bibliometrics combines multiple methods to assess and quantify research output, impact, and developmental trends in scientific studies.^[25]^ Bibliometric analysis involves evaluating measures such as citation patterns, co-authorship networks, cross-national research collaborations, inter-institutional cooperation, journal citation relationships, and shifts in research topics over time. It enables scholars and decision-makers to better understand changing patterns within academic fields, thereby supporting the formulation of research priorities and the distribution of resources. Despite substantial progress in studies on CBT-related depression over the past 2 decades, bibliometric investigations in this area are still limited. Accordingly, the present study seeks to perform a bibliometric evaluation of CBT-related depression research using tools such as VOSviewer, Pajek, and CiteSpace. The aim is to explore developmental patterns and emerging hotspots during the previous twenty years, thereby helping scholars and decision-makers better understand ongoing changes across this research domain. The findings may further support informed decisions on research planning and the distribution of resources.

## 2. Materials and methods

### 2.1. Search strategy and data retrieval

The dataset used for this bibliometric study was retrieved from the Web of Science Core Collection (WOSCC). Due to its comprehensive coverage and standardized structure, WOSCC is widely utilized in scholarly research.^[26]^ Our search strategy uses “TS= (‘Depression’ OR ‘Emotional Depression’ OR ‘Depression, Emotional’ OR ‘Depressive Disorder’ OR ‘Depressive Disorders’ OR ‘Disorder, Depressive’ OR ‘Disorders, Depressive’ OR ‘Neurosis, Depressive’ OR ‘Depressive Neuroses’ OR ‘Depressive Neurosis’ OR ‘Neuroses, Depressive’ OR ‘Depression, Endogenous’ OR ‘Depressions, Endogenous’ OR ‘Endogenous Depression’ OR ‘Endogenous Depressions’ OR ‘Melancholia’ OR ‘Melancholias’ OR ‘Unipolar Depression’ OR ‘Depression, Unipolar’ OR ‘Depressions, Unipolar’ OR ‘Unipolar Depressions’ OR ‘Depressive Syndrome’ OR ‘Depressive Syndromes’ OR ‘Syndrome, Depressive’ OR ‘Syndromes, Depressive’ OR ‘Depression, Neurotic’ OR ‘Depressions, Neurotic’ OR ‘Neurotic Depression’ OR ‘Neurotic Depressions’)” AND TS= (“Behavioral Therapies, Cognitive” OR “Behavioral Therapy, Cognitive” OR “Cognitive Behavioral Therapies” OR “Therapies, Cognitive Behavioral” OR “Therapy, Cognitive Behavioral” OR “Cognition Therapy” OR “Cognition Therapies” OR “Therapies, Cognition” OR “Therapy, Cognitive Behavior” OR “Behavior Therapies, Cognitive” OR “Cognitive Behavior Therapies” OR “Therapies, Cognitive Behavior” OR “Therapy, Cognition” OR “Behavior Therapy, Cognitive” OR “Cognitive Behavior Therapy” OR “Cognitive Psychotherapy” OR “Cognitive Psychotherapies” OR “Psychotherapies, Cognitive” OR “Psychotherapy, Cognitive” OR “Therapy, Cognitive” OR “Cognitive Therapies” OR “Therapies, Cognitive” OR “Cognitive Behaviour Therapy” OR “Behaviour Therapies, Cognitive” OR “Behaviour Therapy, Cognitive” OR “Cognitive Behaviour Therapies” OR “Therapies, Cognitive Behaviour” OR “Therapy, Cognitive Behaviour” OR “CT”). The search was last updated on July 2, 2024. Publications included in the analysis spanned from January 1, 2005, to July 2, 2024. In total, 6384 records, limited to English-language articles and reviews, were selected. All records were subsequently exported in plain text format for further analysis. The search strategy was designed to prioritize sensitivity in order to comprehensively capture the research output related to CBT in depression.

### 2.2. Statistical analysis and visualization of data

For visual mapping and analysis, CiteSpace (version 6.3.R3 Advanced),^[27]^ VOSviewer,^[28]^ and Pajek^[29]^ were applied in this study. Essential information – such as titles, authors, affiliations, countries or regions, journals, keywords, and references – was collected to generate visualizations of collaborative networks among countries, institutions, and authors. Additionally, we constructed keyword co-occurrence networks and keyword timelines, and performed co-citation analyses for authors, journals, and references. The processes of data aggregation, structuring, and visualization were carried out using Microsoft 365 Excel, which facilitated efficient management and organization of the dataset. This study was formally reviewed and granted approval by the Ethics Committee of Guangzhou Civil Affairs Bureau Psychiatric Hospital. As all data were obtained from publicly accessible databases and did not involve identifiable human participants, the requirement for ethical approval and informed consent was waived.

## 3. Results

### 3.1. Retrieval diagram, annual publications, and citations

**Figure 1.**
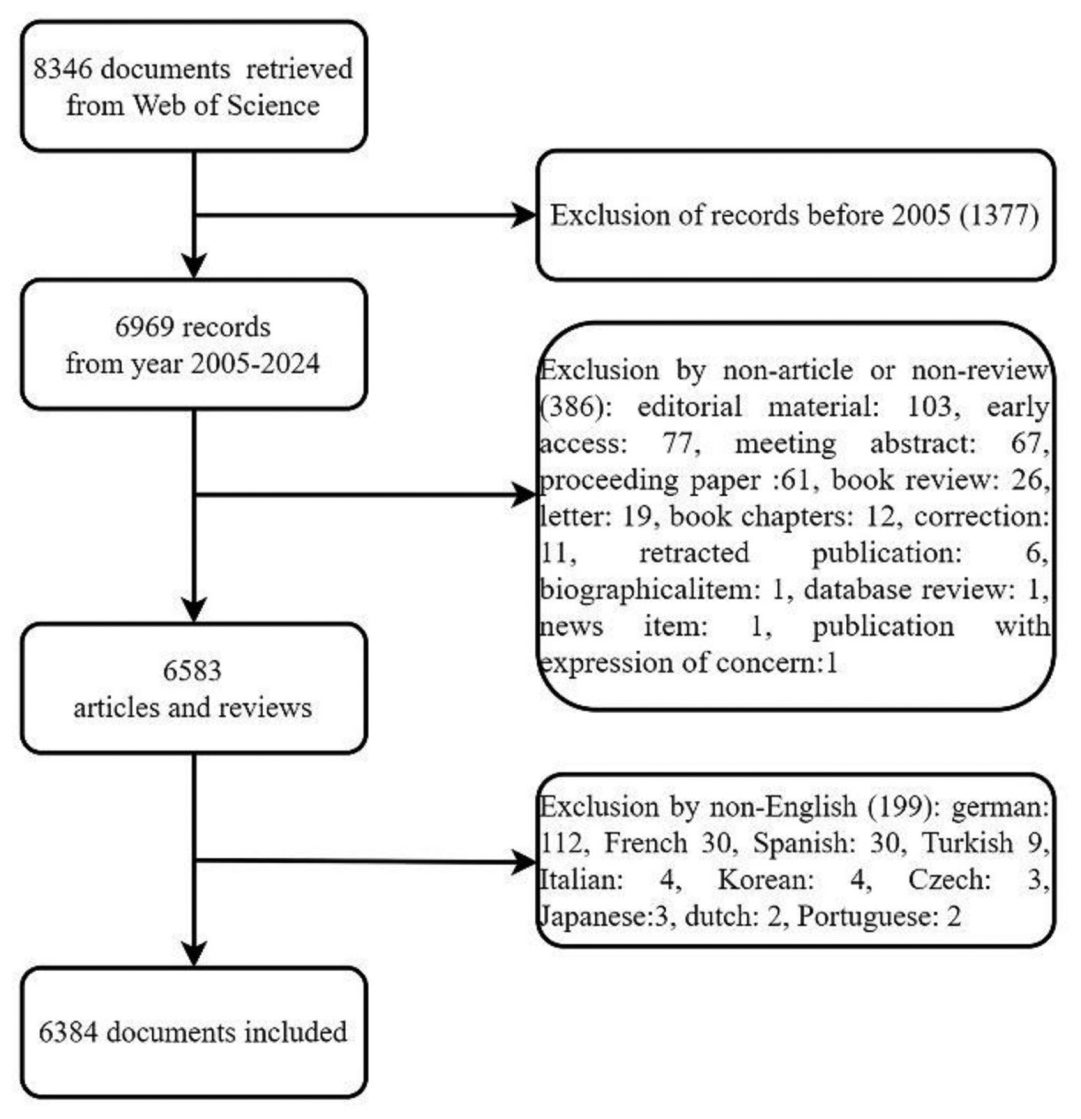
Summary of the literature retrieval and screening process. After applying year, document type, and language restrictions, 6384 articles and reviews were included in the bibliometric analysis.

**Figure 2.**
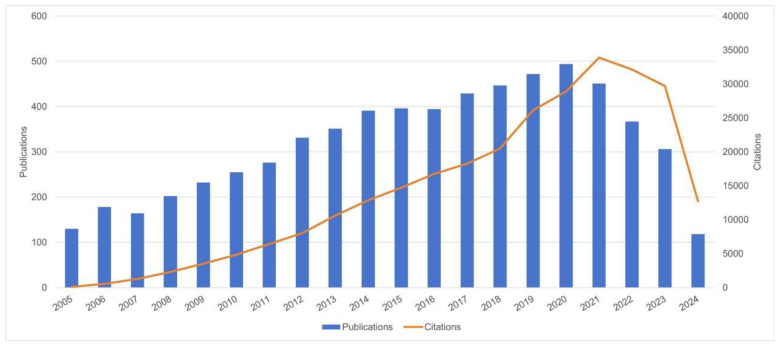
Annual publication counts and citation frequencies of studies on cognitive behavioral therapy for depression over the study period.

A total of 8346 records were retrieved from searches conducted between 2005 and 2024, focusing on English-language articles and reviews. Ultimately, 6384 documents were included in the analysis (Fig. 1). Figure 2 depicts the annual trends in publications and citation frequencies of articles related to CBT for depression from 2005 to 2024. The annual publication count exhibits a steady upward trend with minor fluctuations, peaking at 494 publications in 2020, followed by a sustained decline. In contrast, the citation frequency of articles focused on CBT for depression demonstrates a consistent annual increase, reaching its zenith at 33,882 citations in 2021 before gradually decreasing in subsequent years.

### 3.2. Distribution of countries or regions

Research on CBT-related depression encompasses publications from a total of 86 countries and regions worldwide. Table 1 presents the ten most productive countries based on the number of publications, total citations, and mean citations per article. The United States leads with 2217 publications, followed by the United Kingdom with 1360, while all other countries produced fewer than 1000 articles each. In terms of total citations, the USA ranks first with 121,936, with the United Kingdom second at 80,309; citation counts for other countries remain below 50,000. Regarding mean citations per article, Luxembourg attains the highest value at 146.00, followed by Iraq (117.33), Mexico (109.56), Bahrain (108.00), and Tanzania (106.00). All remaining countries report average citations below 100 per article.

**Table 1:** Top 10 countries or regions by publication count and citation frequency.

Rank	Countries/Regions	Publications	Country/Regions	Citations	Country/Regions	Citations per article
1	USA	2217	USA	121,936	Luxembourg	146.00
2	United Kingdom	1360	United Kingdom	80,309	Iraq	117.33
3	Australia	871	Netherlands	40,150	Mexico	109.56
4	Netherlands	609	Australia	36,149	Bahrain	108.00
5	Canada	560	Sweden	22,410	Tanzania	106.00
6	Germany	462	Canada	21,581	Slovenia	89.00
7	Sweden	417	Germany	20,258	Chile	85.30
8	China	285	North Ireland	10,294	Cyprus	84.33
9	Spain	189	Switzerland	7878	Hungary	77.33
10	Norway	165	Denmark	7050	Netherlands	65.93

USA = United States.

**Figure 3.**
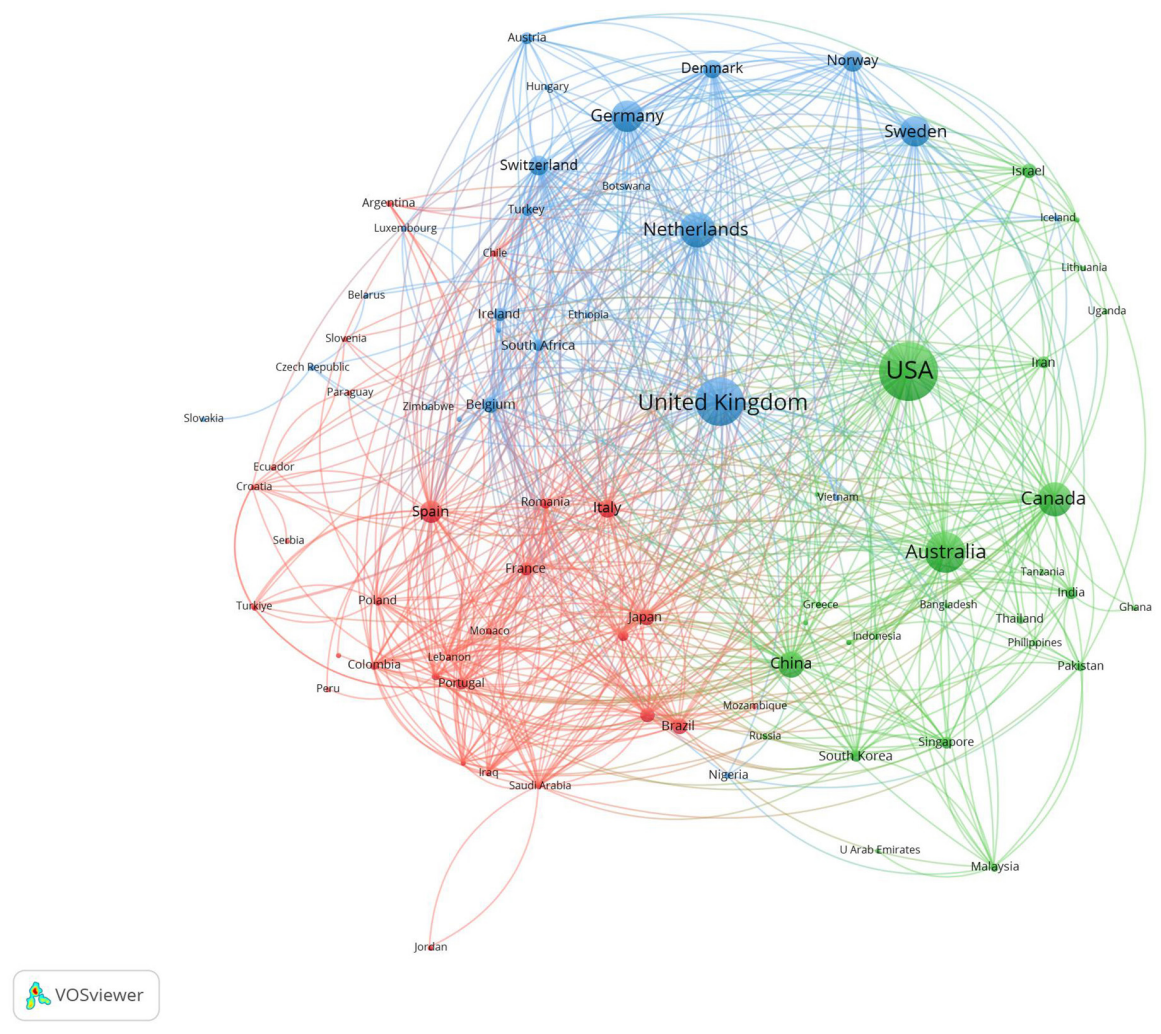
Network visualization of collaborations among countries and regions. Node size represents publication volume, link thickness indicates collaboration strength, and colors denote different collaboration clusters.

Figure 3 depicts the collaborative patterns among countries and regions conducting research on CBT-related depression. Using VOSviewer, these countries were grouped into clusters according to the strength of their collaborations, with each cluster represented by a distinct color. For example, the red cluster comprises Spain, Italy, Brazil, Japan, and several additional countries. Similarly, the green cluster encompasses the USA, Australia, Canada, China, and numerous additional countries. Likewise, the blue cluster involves countries like the United Kingdom, Netherlands, Germany, Sweden, and various others. In Figure 3, the size of each node reflects the number of publications, with the largest nodes corresponding to countries such as the USA, United Kingdom, Canada, Australia, and China. The thickness of the links represents the intensity of collaborations between countries. These results reveal particularly strong connections involving the USA and nations like China, Australia, and Canada, underscoring their prominent roles in CBT-related depression research.

### 3.3. Distribution of institutions

Research on CBT-related depression involves 5012 affiliations. Table 2 presents the ten leading affiliations according to their number of publications and citation performance. Top in terms of publication volume is the Karolinska Institute with 308 articles, followed closely by King’s College London with 263, University of Pennsylvania with 239, Vrije Universiteit Amsterdam with 235, and University of Oxford with 217. Linköping University has 212 publications, while other affiliations have fewer than 200 articles. The University of Oxford ranks first in citations with 22,578, followed by Vrije Universiteit Amsterdam with 20,815, Karolinska Institute with 17,218, University of Pennsylvania with 15,574, and Linköping University with 15,336. Other affiliations have fewer than 15,000 citations.

**Table 2:** The top 10 institutions by publication volume and citation frequency.

Rank	Institutions	Publications	Institutions	Citations
1	Karolinska Institute (Sweden)	308	University of Oxford (United Kingdom)	22,578
2	King’s College London (United Kingdom)	263	Vrije Universiteit Amsterdam (Netherlands)	20,815
3	University of Pennsylvania (USA)	239	Karolinska Institutet (Sweden)	17,218
4	Vrije Universiteit Amsterdam (Netherlands)	235	University of Pennsylvania (USA)	15,574
5	University of Oxford (United Kingdom)	217	Linköping University (Sweden)	15,336
6	Linköping University (Sweden)	212	King’s College London (United Kingdom)	14,129
7	University College London (UCL) (United Kingdom)	149	University of Louisville (USA)	9572
8	University of Manchester (United Kingdom)	149	University of Cincinnati (USA)	9354
9	University of Toronto (Canada)	128	Queen’s University Belfast (United Kingdom)	8844
10	Macquarie University (Australia)	126	University College London (UCL) (United Kingdom)	7616

USA = United States.

**Figure 4.**
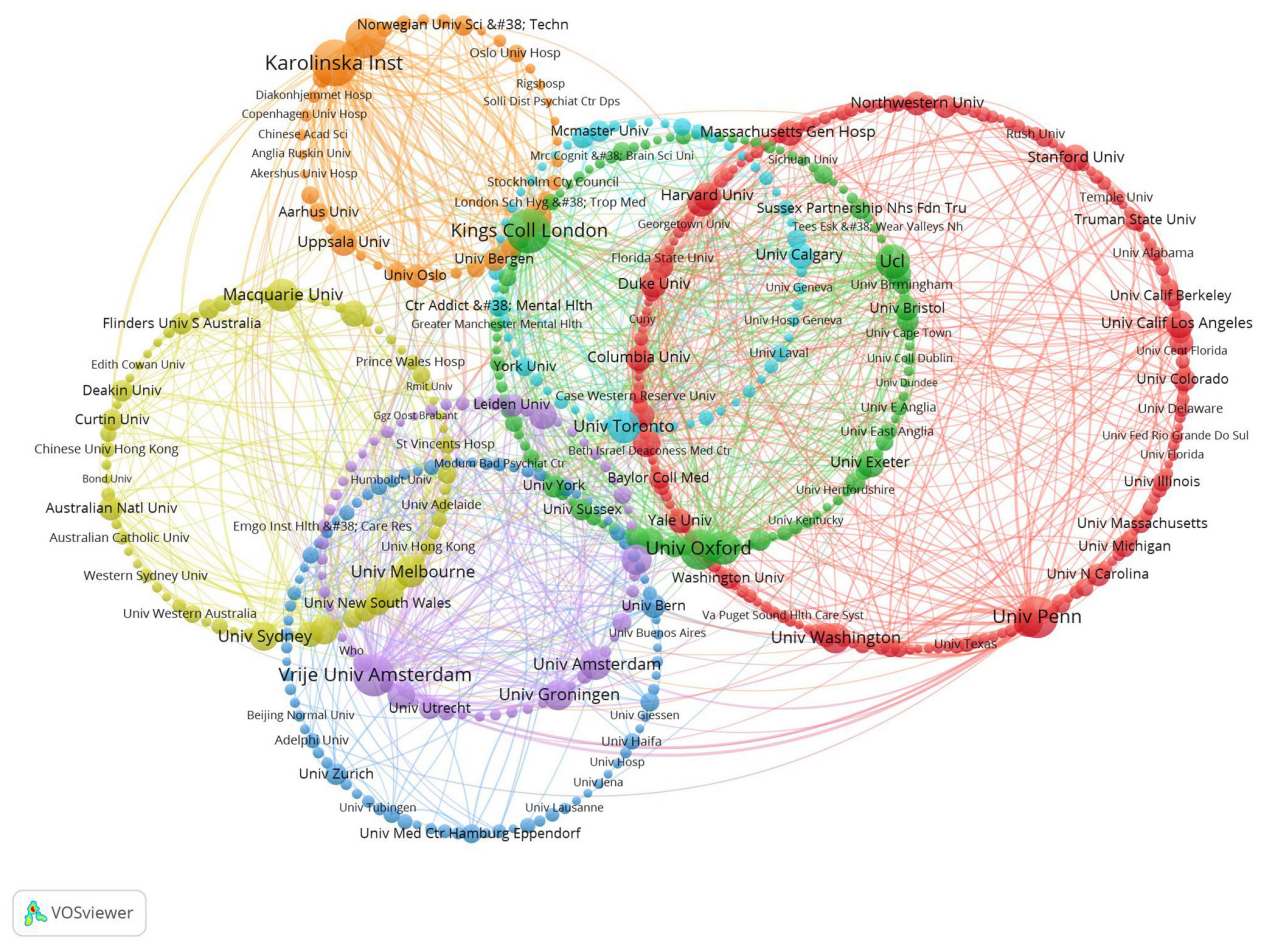
Institutions are grouped into clusters based on collaboration intensity. Node size reflects publication volume, and link thickness represents the strength of institutional collaboration.

Figure 4 presents the network of institutions engaged in research on CBT-related depression. Using VOSviewer, these institutions are organized into 7 clusters, with each cluster represented by a distinct color reflecting the intensity of collaboration. The red cluster includes institutions such as the University of Pennsylvania, the University of California, Los Angeles, and the University of Pittsburgh. The green circle comprises King’s College London, University of Oxford, and University College London. The blue circle features University of Bern, University of Zurich, and University Medical Center Hamburg-Eppendorf. The yellow circle includes University of Melbourne, Macquarie University, and University of Queensland. The purple circle encompasses Vrije Universiteit Amsterdam, University of Amsterdam, and University of Groningen. The light blue cluster includes University of Toronto, McMaster University, and University of Calgary. The orange circle includes Karolinska Institute, Linköping University, and Stockholm University.

These institutions are also pivotal nodes within their respective circles, exhibiting the strongest collaborative ties. Node sizes correspond to publication volumes, highlighting larger nodes such as the University of Pennsylvania, King’s College London, Karolinska Institute, Vrije Universiteit Amsterdam, and the University of Oxford, which have contributed notably to this research domain.

### 3.4. Distribution of authors

Examining authors with the highest publication numbers and co-citation counts in CBT-related depression research provides an overview of researchers and publication trends in this field. In total, 21,645 authors have contributed to this area of study. Table 3 lists the ten authors with the largest number of publications and highest citation frequencies. Andersson, Gerhard tops the list with 170 publications, followed by Cuijpers, Pim with 121 publications; the remaining authors each have fewer than 100 publications. Regarding citation frequency, Cuijpers, Pim holds the highest rank with 14,710 citations, with Andersson, Gerhard in second place at 12,515 citations; All remaining authors have accumulated fewer than 10,000 citations.

**Table 3:** The top 10 authors by publication volume and citation frequency.

Rank	Author	Publications	Author	Citations
1	Andersson, Gerhard	170	Cuijpers, Pim	14,710
2	Cuijpers, Pim	121	Andersson, Gerhard	12,515
3	Carlbring, Per	75	Carlbring, Per	5679
4	Hollon, Steven D.	70	Hollon, Steven D.	5547
5	Thase, Michael E.	60	Van Straten, Annemieke	5018
6	Derubeis, Robert J.	56	Kuyken, Willem	4597
7	Titov, Nickolai	53	Riper, Heleen	4461
8	Kuyken, Willem	52	Derubeis, Robert J.	3294
9	Dear, Blake F.	50	Titov, Nickolai	3079
10	Jarrett, Robin B.	50	Dimidjian, Sona	2859

**Figure 5.**
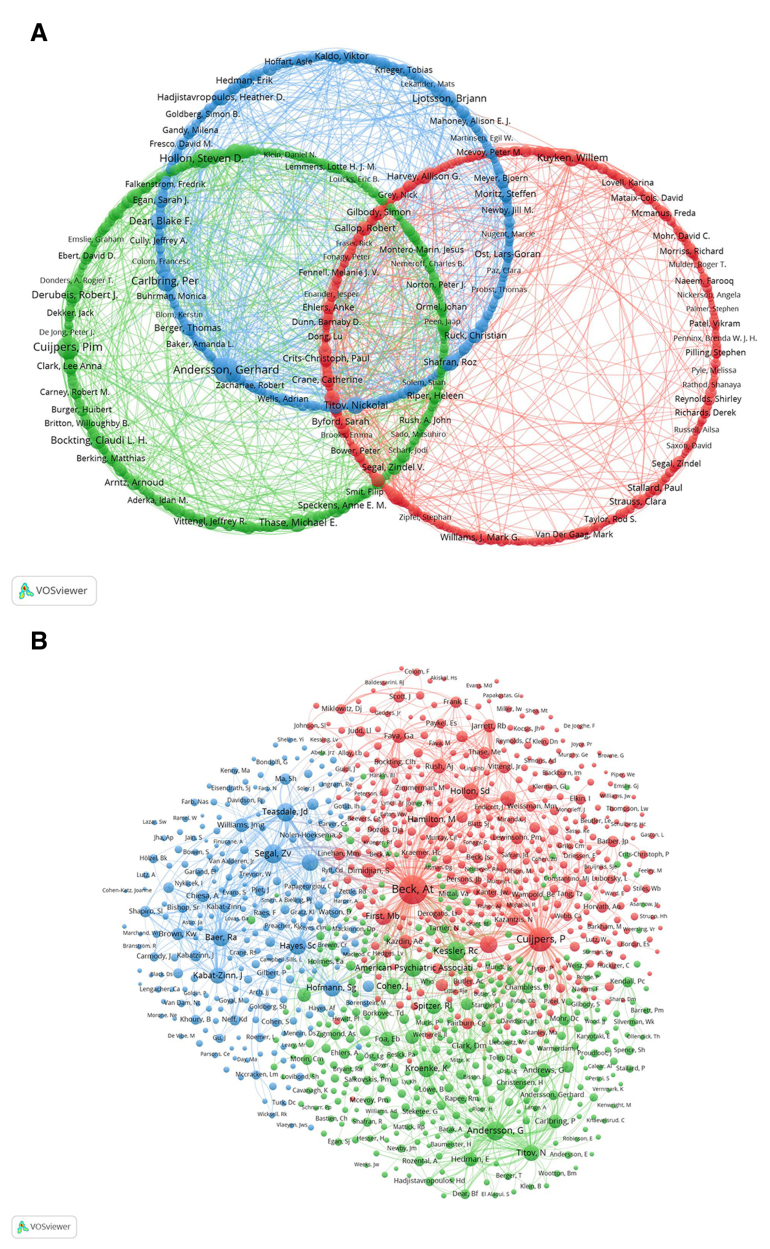
(A) Author collaboration network showing cooperative relationships, with node size representing publication volume and link thickness indicating collaboration strength. (B) Author co-citation network, with node size reflecting co-citation frequency and colors denoting different clusters.

Figure 5A shows the network of authors conducting research on CBT-based depression. Using VOSviewer, the authors are organized into 3 separate clusters according to the intensity of their collaborations, with each cluster represented by a different color. The red circle includes authors such as Byford Sarah, Gilbody Simon, Kuyken Willem, Barkham Michael, Bower Peter, and Lovell Karina, among others; the green circle includes Cuijpers Pim, Hollon Steven D, Bockting Claudi L. H, Thase Michael E, Derubeis Robert J, and Jarrett Robin B, among others. The blue circle comprises Andersson Gerhard, Carlbring Per, Titov Nickolai, Dear Blake F, Ljotsson Brjann, and Lindefors Nils, among others. Node size reflects each author’s publication volume, and line thickness indicates the strength of collaborative ties. Authors with large publication volumes include Byford Sarah, Cuijpers Pim, and Andersson Gerhard.

Figure 5B shows the co-citation network among authors, analyzed with VOSviewer. The authors are organized into 3 main clusters according to the strength of their co-citation links, with each cluster represented by a different color. The red cluster includes authors such as Beck At, Cuijpers P, and Hamilton M. The green cluster features authors such as Andersson G, Cohen J, and Kroenke K. The blue cluster comprises authors like Baer Ra, Hayes Sc, and Segal Zv. The size of each cluster represents the co-citation frequency of the authors within it, with larger circles indicating higher co-citation counts. These authors have the highest co-citation numbers within their respective clusters.

### 3.5. Distribution of journals

Research on CBT-related depression appears in 883 journals, as shown in Table 4, which lists the ten journals with the highest publication numbers and citation counts. The Journal of Affective Disorders ranks first in publication volume with 275 articles, followed by Behavior Research and Therapy with 227 articles, and Mindfulness with 204 articles; all other journals contributed fewer than 200 publications. Among the most cited journals are Behavior Research and Therapy with 18,371 citations, Clinical Psychology Review with 15,502 citations, and Cochrane Database of Systematic Reviews with 15,265 citations; The remaining journals collectively accounted for fewer than 15,000 citations.

**Table 4:** The top 10 journals by publication volume and citation frequency.

Rank	Journals	Publications	Journals	Co-citations
1	Journal of Affective Disorders	275	Behavior Research and Therapy	18,371
2	Behavior Research and Therapy	227	Clinical Psychology Review	15,502
3	Mindfulness	204	Cochrane Database of Systematic Reviews	15,265
4	Journal of Consulting and Clinical Psychology	158	Journal of Consulting and Clinical Psychology	13,620
5	Behavioral and Cognitive Psychotherapy	128	Journal of Affective Disorders	9819
6	Psychotherapy Research	127	Psychological Medicine	8471
7	Cognitive Therapy and Research	125	Plops One	5261
8	BMC Psychiatry	112	Depression and Anxiety	5229
9	Psychological Medicine	101	Mindfulness	4878
10	Behavior Therapy	95	Journal of Medical Internet Research	4802

**Figure 6.**
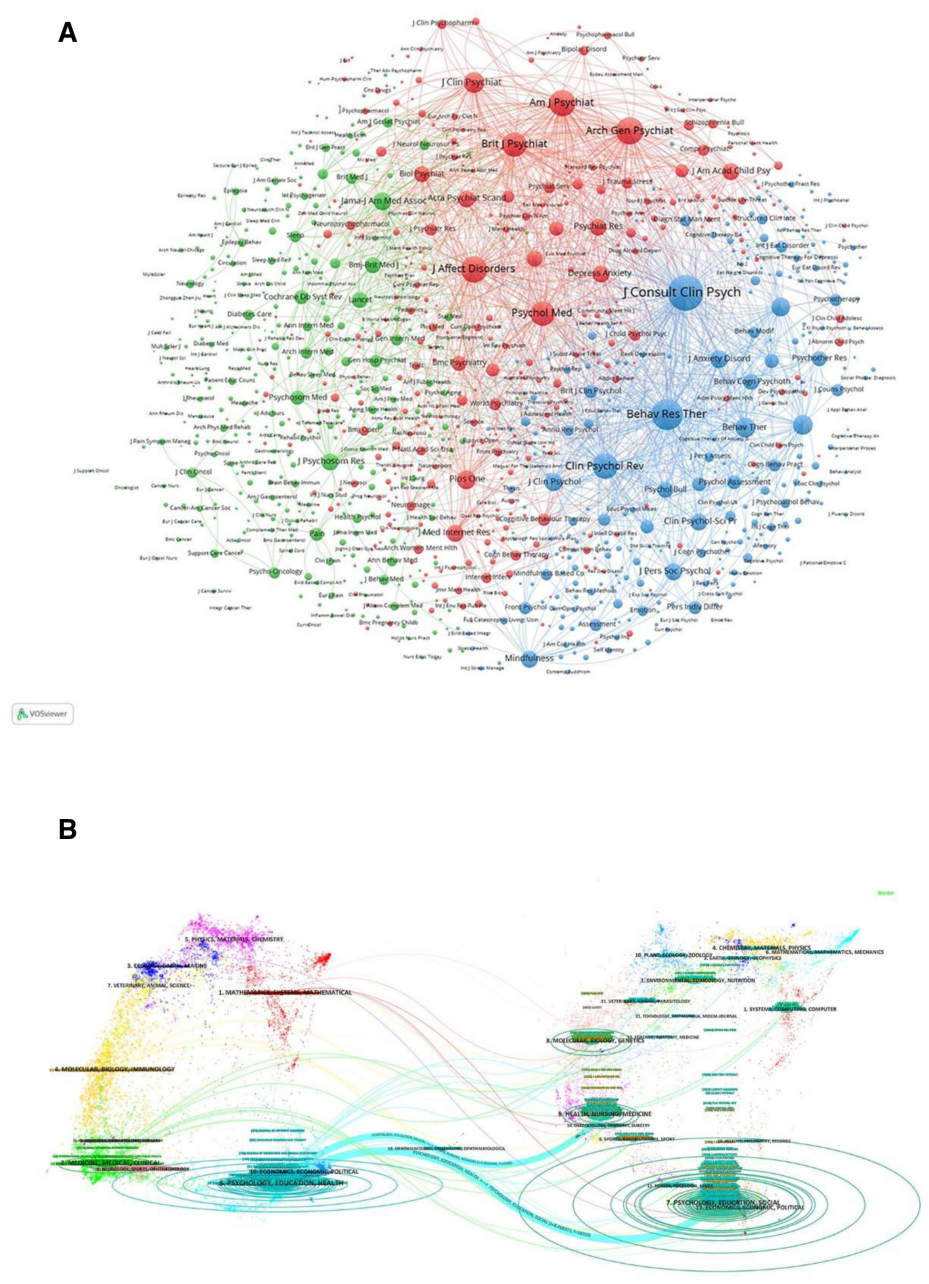
(A) Network of journals publishing CBT-related depression research, clustered by citation relationships into 3 color-coded groups: red (Journal of Affective Disorders, American Journal of Psychiatry, Psychological Medicine), green (JAMA – Journal of the American Medical Association, Journal of Psychosomatic Research, The Lancet), and blue (Journal of Consulting and Clinical Psychology, Behaviour Research and Therapy, Clinical Psychology Review), as analyzed using VOSviewer. (B) Dual-map overlay showing citation pathways between citing (left) and cited (right) journals, illustrating the flow of knowledge from foundational to frontier research areas.

Figure 6A presents the network of journals that publish research on CBT-related depression, along with their interconnections, analyzed with VOSviewer. The journals are divided into 3 clusters, each represented by a different color to indicate thematic relationships. The red cluster includes journals such as the Journal of Affective Disorders, the American Journal of Psychiatry, and Psychological Medicine. The green cluster includes JAMA – Journal of the American Medical Association, Journal of Psychosomatic Research, and The Lancet. The blue cluster encompasses Journal of Consulting and Clinical Psychology, Behaviour Research and Therapy, and Clinical Psychology Review.

Knowledge flow analysis was conducted to examine the development of citation and co-citation patterns among journals that cite and are cited by each other.^[30]^ The dual-map overlay of journals visualizes the dissemination of topics, citation pathways, and changes in research focal points across academic journals.^[30,31]^ In the Dual-map, labels on the left indicate citing journals, while labels on the right indicate cited journals. The colored curves connecting the citing and cited maps represent the overall citation relationships. In the citing map, the vertical axis of each ellipse increases with the number of publications for a journal, whereas the horizontal axis extends with the number of contributing authors. Citing journals mainly cover areas such as PSYCHOLOGY, EDUCATION, HEALTH, ECONOMICS, ECONOMIC, POLITICAL, MEDICINE, MEDICAL, CLINICAL, NEUROLOGY, SPORTS, and OPHYRALMOLOGY, reflecting current research activity. Cited journals focus on topics including PSYCHOLOGY, EDUCATION, SOCIAL, ECONOMICS, ECONOMIC, POLITICAL, HEALTH, NURSING, and MEDICINE, providing the foundational knowledge base as shown in Figure 6B.

### 3.6. Analysis of keywords

**Figure 7.**
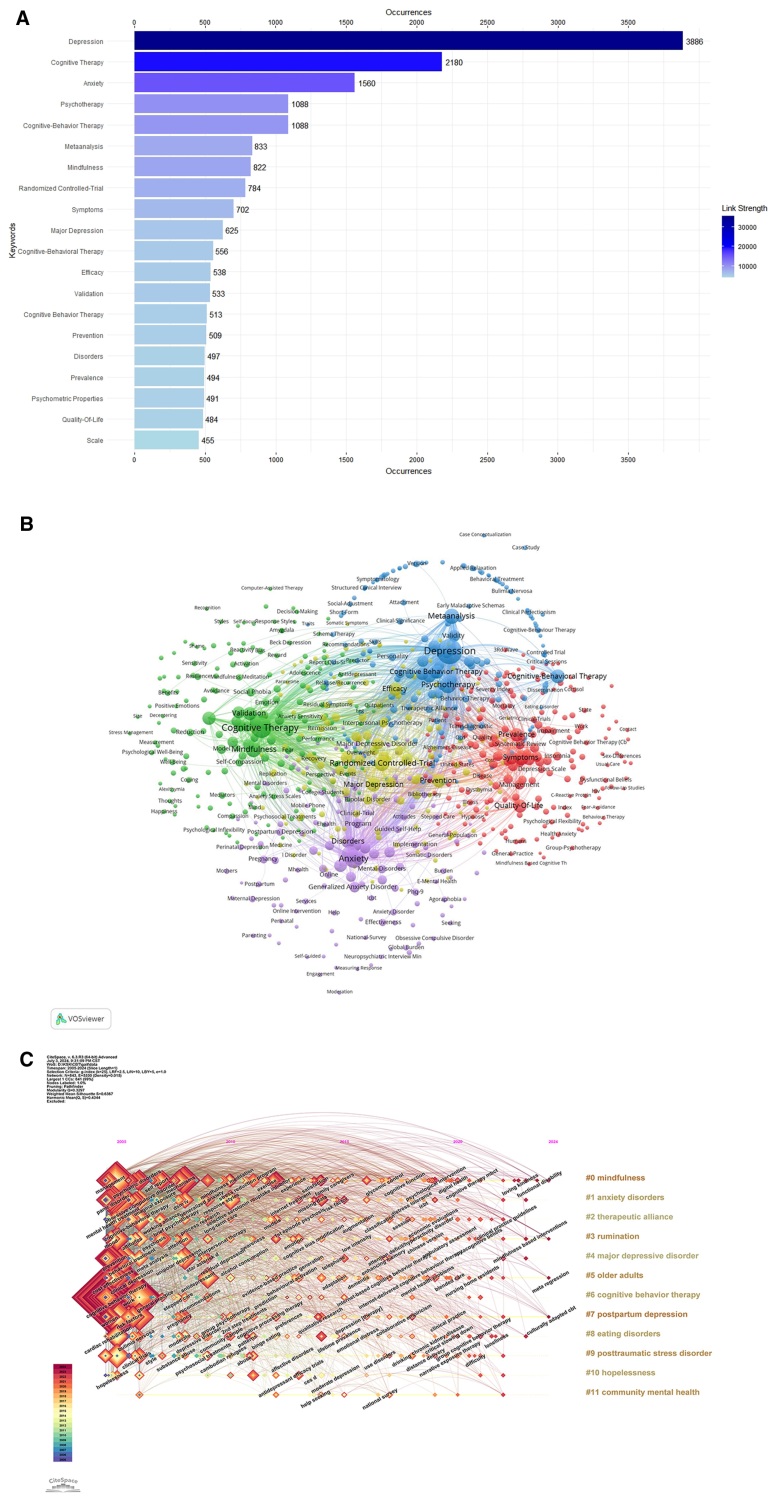
(A) The 20 most frequent keywords in CBT-related depression research, led by depression, cognitive therapy, anxiety, psychotherapy, cognitive-behavior therapy, and meta-analysis; all other keywords appear fewer than 450 times. Blue color intensity indicates keyword frequency, with darker blue representing higher occurrence. (B) Network of keyword co-occurrences, with closely related keywords grouped into 5 color-coded clusters. Red: symptoms, prevalence, quality-of-life, intervention; green: cognitive therapy, mindfulness, validation, psychometric properties; blue: depression, psychotherapy, meta-analysis, cognitive-behavioral therapy; yellow: randomized controlled-trial, major depression, efficacy, prevention; purple: anxiety, cognitive-behavior therapy, disorders, interventions. (C) Chronological visualization of keyword emergence, where square size reflects the frequency of subsequent occurrences. Keywords on the right correspond to the thematic clusters shown on the left.

Keywords are essential for exploring the forefront of research in CBT-related depression. Figure 7A showcases the 20 most frequently mentioned keywords. Leading the list is depression, followed by CT, anxiety, psychotherapy, cognitive-behavior therapy, meta-analysis, mindfulness, randomized controlled-trial, symptoms, major depression, cognitive-behavioral therapy, efficacy, validation, cognitive behavior therapy, prevention, disorders, prevalence, psychometric properties, quality-of-life, and scale. All other keywords appear fewer than 450 times.

Figure 7B presents a keyword co-occurrence network, in which closely related keywords are organized into 5 clusters, each represented by a different color. The red cluster contains terms such as symptoms, prevalence, quality-of-life, and intervention. The green cluster features terms such as CT, mindfulness, validation, and psychometric properties. The blue cluster includes keywords like depression, psychotherapy, metaanalysis, and cognitive-behavioral therapy. The yellow cluster comprises randomized controlled-trial, major depression, efficacy, and prevention. The purple cluster includes anxiety, cognitive-behavior therapy, disorders, and interventions.

Figure 7C provides a visual representation of the temporal emergence of these keywords, with the size of each square reflecting the frequency of later occurrences across the dataset. The keywords displayed in the right column correspond to the thematic clusters identified on the left side of the figure, facilitating interpretation of the relationships between clusters and terms.

### 3.7. Analysis of reference

Table 5 presents the 15 articles^[32–46]^ with the highest citation counts, with the most cited article being “Cognitive-behavioral interventions for children who have been sexually abused,”^[32]^ which has received 8751 citations. This review^[32]^ examines the effectiveness of CBT in addressing the immediate and long-term impacts of childhood sexual abuse on children and adolescents up to 18 years old. Analyzing 10 trials involving 847 participants, the study suggests that CBT interventions, typically involving children and a non-offending parent, may positively impact symptoms such as depression, post-traumatic stress disorder (PTSD), and anxiety. However, these effects are generally moderate and often not statistically significant across all outcomes. The review underscores the need for improved study quality and more rigorous trials to strengthen the evidence supporting CBT for children who have experienced sexual abuse. Ranked second in citation frequency, the article titled “ACT: Model, processes and outcomes”^[33]^ has been cited 3484 times. This article presents and reviews acceptance and commitment therapy (ACT), focusing on its model of psychopathology and treatment. ACT stands out for its integration with Relational Frame Theory, a robust research program on human language and cognition. This connection reflects earlier behavior therapy principles grounded in fundamental behavioral concepts. The evidence supporting ACT – through correlational, component, process of change, and outcome comparisons – is promising but not yet fully mature. ACT appears to operate through mechanisms distinct from traditional CBT. However, more well-controlled studies are needed to conclusively determine whether ACT is generally more effective than other active treatments across diverse conditions. The remaining articles in the top 15 by citation count each accumulated fewer than 2000 citations, showing a clear difference from the most cited article.

**Table 5:** The top 15 articles by citation frequency.

Rank	Article title	Authors/year/reference number	Source title	Citations	DOI	Document type
1	Cognitive-behavioral interventions for children who have been sexually abused	MacDonald, G et al 2012,^[32]^	Cochrane Database of Systematic Reviews	8751	10.1002/14651858.CD001930.pub3	Review
2	Acceptance and commitment therapy: Model, processes and outcomes	Hayes, SC et al 2006,^[33]^	Behavior Research and Therapy	3484	10.1016/j.brat.2005.06.006	Review
3	The empirical status of cognitive-behavioral therapy: A review of meta-analyses	Butler, AC et al 2006,^[34]^	Clinical Psychology Review	1918	10.1016/j.cpr.2005.07.003	Review
4	Mindfulness-based therapy: A comprehensive meta-analysis	Khoury, B et al 2013,^[35]^	Clinical Psychology Review	1216	10.1016/j.cpr.2013.05.005	Article
5	Internet-based cognitive behavior therapy for symptoms of depression and anxiety: a meta-analysis	Spek, V et al 2006,^[36]^	Psychological Medicine	1082	10.1017/S0033291706008944	Review
6	Positive psychology interventions: a meta-analysis of randomized controlled studies	Bolier, L et al 2013,^[37]^	BMC Public Health	1054	10.1186/1471-2458-13-119	Article
7	WHO World Mental Health Surveys International College Student Project: Prevalence and Distribution of Mental Disorders	Auerbach, RP et al 2018,^[38]^	Journal of Abnormal Psychology	1016	10.1037/abn0000362	Article
8	Randomized trial of behavioral activation, cognitive therapy, and antidepressant medication in the acute treatment of adults with major depression	Dimidjian, S et al 2006,^[39]^	Journal of Consulting and Clinical Psychology	995	10.1037/0022-006X.74.4.658	Article
9	How do mindfulness-based cognitive therapy and mindfulness-based stress reduction improve mental health and wellbeing? A systematic review and meta-analysis of mediation studies	Gu, J et al 2015,^[40]^	Clinical Psychology Review	986	10.1016/j.cpr.2015.01.006	Review
10	Comorbidity: A network perspective	Cramer, AOJ et al 2010,^[41]^	Behavioral and Brain Sciences	891	10.1017/S0140525X09991567	Article
11	Mindfulness-based stress reduction for healthy individuals: A meta-analysis	Khoury, B et al 2015,^[42]^	Journal of Psychosomatic Research	825	10.1016/j.jpsychores.2015.03.009	Review
12	Intrusive Images in Psychological Disorders: Characteristics, Neural Mechanisms, and Treatment Implications	Brewin, CR et al 2010,^[43]^	Psychological Review	751	10.1037/a0018113	Review
13	Computer Therapy for the Anxiety and Depressive Disorders Is Effective, Acceptable and Practical Health Care: A Meta-Analysis	Andrews, G et al 2010,^[44]^	PLOS ONE	724	10.1371/journal.pone.0013196	Article
14	Behavioral activation treatments of depression: A meta-analysis	Cuijpers, P et al 2007,^[45]^	Clinical Psychology Review	705	10.1016/j.cpr.2006.11.001	Review
15	Guided Internet-based vs face-to-face cognitive behavior therapy for psychiatric and somatic disorders: a systematic review and meta-analysis	Andersson, G et al 2014,^[46]^	World Psychiatry	684	10.1002/wps.20151	Article

**Figure 8.**
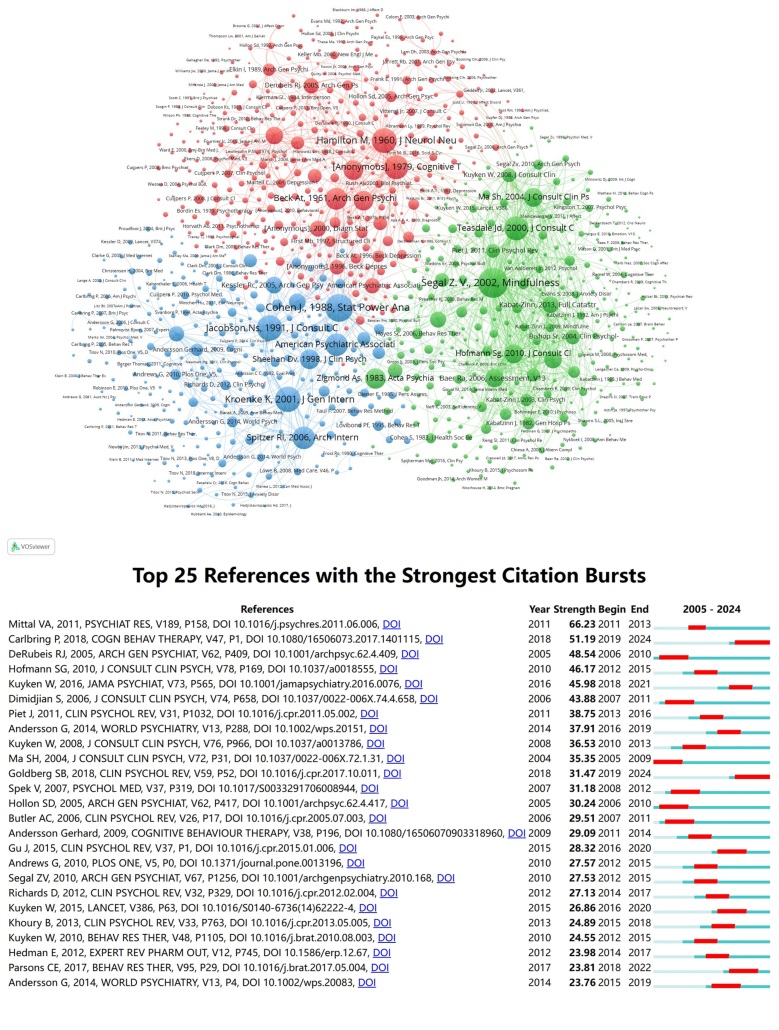
(A) Network of co-cited references grouped into 3 color-coded clusters. Circle size represents the co-citation weight of each reference. (B) Top references with significant citation bursts over time. Each horizontal line represents a reference, and the red segment on the line indicates the start and end years of its citation burst.

Co-citation analysis of references was performed to investigate how studies are interconnected through shared citations. In Figure 8A, these connections are depicted as a network diagram, with studies grouped into 3 major clusters, each represented by a distinct color. In the red cluster, foundational references include Hamilton M 1960,^[47]^ Dimidjian S 2006,^[39]^ and Beck AT 1961.^[48]^ The green cluster consists of references such as Hofmann Sg 2010,^[49]^ Teasdale JD 2000,^[50]^ and Ma SH 2004.^[51]^ References within the blue cluster include Spitzer RL 2006,^[52]^ Jacobson NS 1991,^[53]^ and Kroenke K 2001.^[54]^ In the figure, the diameter of each circle represents its co-citation strength, indicating the relative influence of these references within their respective clusters, which are color-coded for clarity.

Figure 8B presents the 25 references showing the strongest citation bursts, with the earliest burst recorded in 2005. Featured is the study “Mindfulness-based CT for depression: replication and exploration of differential relapse prevention effects” by Ma SH et al,^[51]^ published in 2004 in the Journal of Consulting and Clinical Psychology. Mittal VA et al’s paper^[55]^ on “Diagnostic and Statistical Manual of Mental Disorders” in psychiatry research attained the highest citation burst intensity of 66.23 in 2011. In 2012, 4 citation bursts were observed, marking a key year.

## 4. Discussion

This bibliometric study of CBT in depression utilized VOSviewer, Pajek, and CiteSpace. The analysis covers the period from January 1, 2005, to July 2, 2024, including contributions from 86 countries or regions, 5012 institutions, and 21,645 authors. Results indicate that publications peaked at 494 papers in 2020, while citations reached a maximum of 33,882 in 2021 (Fig. 2). Both publication and citation numbers declined in the following years, highlighting a trend that requires careful interpretation. First, from a methodological perspective, citation counts for recent publications are naturally lower due to limited accumulation time, which may partly explain the downward trend. Second, the COVID-19 pandemic likely disrupted research activities, delaying clinical trials, data collection, and manuscript preparation in the field of depression and CBT. Third, shifts in research priorities, such as increasing emphasis on digital or remote interventions, may have influenced publication patterns and may not be fully captured by our search strategy focused on traditional CBT. These factors likely interact, and readers should consider them collectively when interpreting the temporal trends observed in our bibliometric analysis.

The analysis of countries and regions underscores the dominant presence of the USA and United Kingdom in the field of CBT for depression, leading in terms of both publication volume and citation frequency. This highlights their significant influence and leadership within this domain (Table 1). Additionally, among the top 10 institutions by publication count, one is located in the USA. Regarding citation frequency, 3 institutions from the USA feature among the top 10. Similarly, 4 institutions from the United Kingdom appear in the top 10 for publication volume, with an equal number also prominent in citation frequency rankings (Table 2). The Karolinska Institute ranks first in publication output with 308 papers, while the University of Oxford has the highest citation count at 22,578. Regarding institutional collaboration, the USA and the United Kingdom occupy central positions, exhibiting extensive interconnections. Moreover, nations like Australia, the Netherlands, Canada, Germany, Sweden, and China, which exhibit substantial publication volumes, also engage actively in collaborations and exchanges with various other countries and regions (Fig. 3).

Andersson Gerhard occupies a prominent role in CBT and depression research, ranking among the top authors in publication volume and holding the second-highest citation count (Table 3). Cuijpers Pim, Carlbring Per, Hollon Steven D, and Andersson Gerhard each rank within the top 4 for both publication volume and citation frequency. Moreover, Andersson Gerhard and Cuijpers Pim have publication volumes and citation frequencies at least 35% and 50% higher than other authors, respectively. Andersson Gerhard occupies a prominent position in both total publication volume and citation frequency. Additionally, studies by Andrews G et al, including “Computer Therapy for Anxiety and Depressive Disorders Is Effective, Acceptable and Practical Health Care: A Meta-Analysis^[44]^” and “Guided Internet-based vs face-to-face cognitive behavior therapy for psychiatric and somatic disorders: a systematic review and meta-analysis^[46]^” rank 13th and 15th in citation frequency. Cuijpers P et al’s “Behavioral activation treatments of depression: A meta-analysis^[45]^” ranks 14th in citation frequency. Dimidjian Sona is ranked 10th in terms of citation frequency, whereas Dimidjian S et al’s study^[39]^ titled “Randomized trial of behavioral activation, CT, and antidepressant medication in the acute treatment of adults with major depression” holds the 8th position in citation frequency. This highlights the authors’ substantial influence and recognized expertise within the field, reflecting their consistent contributions to advancing research and knowledge in CBT and depression.

We analyzed journals and found that the following 5 journals are not only ranked in the top ten by publication volume but also in citation frequency (Table 4): Journal of Affective Disorders, Behavior Research and Therapy, Mindfulness, Journal of Consulting and Clinical Psychology, and Psychological Medicine. These journals address a spectrum of topics: encompassing affective disorders, treatments, and prevention of mental health disorders, mindfulness practices and their effects, clinical psychology methodologies, and a broad spectrum of issues in psychiatry and psychology, spanning clinical and experimental research, epidemiology, and mental health policy. The focus of these themes corresponds with the patterns observed in the dual-map analysis shown in Figure 6B.

Keyword analysis is an essential component in research on depression and CBT, offering insights into prevailing trends and focal topics. By analyzing commonly occurring keywords, it is possible to identify central themes and key areas of investigation within the field. The keywords analyzed in this study – depression, CT, anxiety, psychotherapy, cognitive-behavior therapy, meta-analysis, mindfulness, randomized controlled trial, symptoms, major depression, cognitive-behavioral therapy, efficacy, validation, cognitive behavior therapy, prevention, disorders, prevalence, psychometric properties, quality-of-life, and scale – illustrate the comprehensive scope of research on the treatment and prevention of depression using CBT (Fig. 7A). These keywords reflect the diverse and broad scope of research conducted within this field.

Keyword analysis is instrumental in elucidating the role and current state of CBT in treating depression. Our research identifies 4 categories of high-frequency keywords, highlighting key areas of focus and interest. The first category is symptom-related keywords, which encompass terms such as “depression,” “anxiety,” “symptoms,” “major depression,” and “disorder,” and pertain to the clinical indications for CBT treatment.^[34]^ Previous researches^[56–59]^ have found that combining psychological interventions with antidepressants, either as alternatives or adjuncts, significantly reduces the risk of relapse compared to the use of antidepressants alone. The second category is treatment-related keywords, which include “CT,” “CBT,” “psychotherapy,” and “mindfulness therapy.” Traditional CBT, when adapted to diverse cultural contexts and integrated with other approaches, has evolved into various forms such as mindfulness-based cognitive therapy (MBCT), dialectical behavior therapy, and mindfulness-based stress reduction. MBCT has demonstrated significant efficacy in managing depressive symptoms, reducing psychological distress, and serving as a preventive intervention or addressing residual depressive symptoms.^[60]^ The third category is research method-related keywords, including “meta-analysis,” “randomized controlled trial,” “effectiveness,” “psychometric properties,” and “validation.” The application of CBT in clinical practice has expanded, making it one of the most utilized psychotherapies. The progression of evidence-based medicine necessitates the validation of CBT’s therapeutic effects.^[61–63]^ Current research consistently supports the robust evidence base of CBT, which is reflected in its inclusion in treatment guidelines. The fourth category is disease management-related keywords, which include “prevention,” “prevalence,” and “quality of life.” The primary goals of treating depression are to improve cure rates, reduce suicide and disability rates, enhance patients’ quality of life, prevent relapse, and effectively restore social functioning.^[64]^ Utilizing CBT in the maintenance treatment of depression can improve treatment adherence and satisfaction, enhance sleep and life quality, reduce negative emotions, and promote disease recovery.^[65]^

Depression exhibits a highly complex pathogenesis with multiple contributing factors. Although the precise mechanisms remain not fully elucidated, its pathogenesis is intricately linked to a constellation of influences, including social environment, psychological factors, and biological factors.^[66]^ Addressing these multifaceted origins of depression requires effective therapeutic interventions, among which CBT is a commonly used, convenient, and effective approach. CBT operates by guiding patients to monitor, identify, and rectify distorted cognitions and maladaptive behaviors related to their issues.^[67–69]^ It is noted for its high safety profile and demonstrated efficacy in reducing depressive symptoms. However, the clinical application of CBT encounters several practical challenges, including a shortage of experienced clinicians, high treatment costs, and substantial travel time for patients residing in remote areas. These obstacles contribute to difficulties in treatment adherence, leading to many patients not receiving consistent and effective psychological therapy.^[70]^

Over the past several decades, multiple forms of CBT have been designed to tackle these challenges.

Among these adaptations, simplified cognitive behavioral therapy (SCBT) emphasizes the gradual and systematic application of CBT’s theoretical framework, including behavioral activation, the identification and modification of negative automatic thoughts, and the development of new core beliefs. Research^[71]^ indicates that SCBT can ameliorate depressive symptoms in patients with anxiety disorders and enhance their overall quality of life. For example, a study^[72]^ conducted during the COVID-19 pandemic demonstrated that SCBT effectively improved insomnia symptoms in women with COVID-19 in mobile cabin hospitals, particularly for acute insomnia associated with stress. While both SCBT and pharmacotherapy can indirectly enhance quality of life by alleviating depressive symptoms, SCBT also directly addresses aspects related to quality of life in individuals with depression. Future advancements could focus on refining treatment manuals and integrating both mandatory and optional course components to provide therapists with greater flexibility in clinical practice.

In addition, internet-delivered cognitive behavioral therapy (ICBT) involves administering CBT interventions via online platforms, utilizing predefined digital programs to communicate treatment goals and tasks, and interacting with patients through computerized systems.^[15]^ ICBT offers several advantages, including anonymity, elimination of time and location constraints, increased accessibility, and greater flexibility in format. These features enhance the efficiency of mental health services while reducing patients’ economic burden and stigma associated with treatment.^[73]^ For instance, a randomized study^[74]^ on MBCT for pregnant and postpartum women demonstrated the feasibility of virtual network-based MBCT in addressing perinatal psychological symptoms. However, the highly structured nature of ICBT may limit the potential for personalized treatment. Research indicates that guided ICBT is more effective than unguided ICBT, particularly benefiting patients with moderate to severe depression.^[75]^ The structured format of ICBT helps mitigate adverse effects that might arise from therapists’ insufficient familiarity with techniques, personal values, or personality issues. Nonetheless, this structured approach can also constrain the provision of individualized, tailored treatment.^[76]^ Future advancements may focus on integrating virtual AI therapists, utilizing portable devices for reminders, and further developing the digitalization of psychotherapy to enhance personalization and effectiveness.

Furthermore, computer-aided cognitive behavioral therapy (CCBT) incorporates various scenario-based videos to assist patients in identifying automatic thoughts and reinforces learning through multimedia resources, including videos, audio recordings, and interactive exercises. Meta-analyses^[77,78]^ have demonstrated that integrating computerized components with clinician support is associated with enhanced improvements in depressive symptoms. Additionally, CCBT has shown a significant effect on reducing depressive symptoms among primary care patients, outperforming standard treatment approaches.^[79]^

Additionally, clinical studies have explored the integration of CBT with various other treatment modalities.^[80]^ For instance, study^[81]^ comparing exposure-based cognitive therapy with standard CBT reported that exposure-based cognitive therapy enhances emotional processing and increases self-efficacy, contributing to improved long-term outcomes in depression. Other studies have examined the comparative effectiveness of CBT and alternative therapies across different populations.^[15,82]^ For instance, IPT has been shown to be as effective as CBT in treating adolescent depression.^[15,82]^ Moreover, both CBT and MBCT have demonstrated efficacy in preventing relapse of major depressive disorder by equipping patients with metacognitive skills to manage distressing thoughts and emotions.^[83]^ While MBCT has shown sustained effectiveness in preventing depression relapse, behavioral activation therapy and interpersonal therapy have exhibited delayed onset effects in depression prevention. Overall, all psychological interventions have proven to be more effective than supportive counseling.^[84]^

Overall, mobile health applications have become increasingly prevalent in the mental health domain, encompassing various platforms such as computers, tablets, smartphones, and smart wearable devices. Advances in virtual reality and augmented reality-based CBT have the potential to introduce innovative treatment modalities, thereby enhancing both the therapeutic experience and efficacy. The progression of CBT-related mobile applications, computer-based virtual reality technologies, and the integration and refinement of artificial intelligence are expected to lead to more effective and interactive personalized digital behavioral health interventions for individuals with depression. In the post-COVID-19 landscape, telehealth has become widespread, with preliminary evidence supporting the effectiveness of both in-person and telehealth-delivered IPT and CBT for treating depression.^[78,85]^ Nevertheless, further research is needed to elucidate the optimal combinations and formats of these interventions. As the field advances, interdisciplinary collaboration and a commitment to evidence-based practice will be essential for maximizing CBT’s potential in addressing the multifaceted challenges of depression.

This study has several strengths. It provides a comprehensive bibliometric analysis of CBT in depression over the past 2 decades, covering 6384 publications. By using multiple bibliometric tools (VOSviewer, CiteSpace, Pajek), the study ensures robust visualization and multidimensional mapping of the research landscape. The identification of influential authors, institutions, journals, and emerging research hotspots offers valuable guidance for future studies and international collaboration.

Nonetheless, some limitations should be noted. First, no formal sensitivity analysis was conducted to examine the impact of alternative search strategies or reduced synonym sets. Although the broad retrieval strategy enhanced the completeness of the dataset and is consistent with common practice in bibliometric research, future studies may benefit from systematically comparing different search strategies to further assess the robustness of the findings. Second, this analysis relied exclusively on the WOSCC and English-language publications, which may introduce structural bias by underrepresenting certain regions, languages, or local journals. As a result, the findings may preferentially reflect research from regions and journals well indexed in WOSCC. Future studies could incorporate additional databases (e.g., Scopus, PubMed, or regional databases) to achieve a more globally representative bibliometric analysis. Finally, it is important to emphasize that bibliometric indicators, including citation counts, centrality measures, and co-occurrence frequencies, primarily reflect patterns of scientific attention rather than clinical or therapeutic efficacy. High citation density or centrality does not necessarily indicate that a study has strong translational impact or proven therapeutic effectiveness. In addition, citation counts for recent publications may be underestimated due to limited accumulation time, which could affect the identification of emerging trends or research hotspots. Furthermore, we did not conduct a formal quality appraisal of the included literature, and variations in study design, sample size, or methodological rigor may influence the interpretation of influential works and hotspots identified in this analysis. Therefore, readers should interpret the bibliometric findings with consideration of these limitations, and recognize that bibliometric prominence does not directly equate to clinical or translational significance.

## 5. Conclusions

This study applied bibliometric methods to examine the development and evolving trends of research on CBT for depression over the past twenty years. Overall, both the number of publications and citation counts have increased, although a modest decrease has been observed in the most recent years. The United States occupies a central role in this research area, with prominent institutions such as the Karolinska Institute, King’s College London, and the University of Pennsylvania leading in contributions. Key authors in this field include Andersson Gerhard, Cuijpers Pim, and Carlbring Per. CBT has demonstrated substantial efficacy in treating depression, with various adaptations enhancing its applicability and effectiveness. SCBT, ICBT, and CCBT address practical challenges and offer new ways to engage patients. The integration of CBT with other therapies and advancements in mobile health technologies further improve treatment accessibility and personalization. Ongoing research and interdisciplinary collaboration will be essential to optimize these approaches and fully leverage CBT’s potential in addressing the complex challenges of depression. This analysis highlights research patterns and emerging directions, offering information useful for identifying key topics and potential collaboration opportunities in the field.

## Acknowledgments

The authors sincerely thank all individuals involved in the careful collection and analysis of data. The authors also appreciate the constructive feedback provided by the reviewers and the editorial board of Medicine.

## Author contributions

**Conceptualization:** Shaokui Kan, Xinying Li, Nuannuan Chen.

**Data curation:** Shaokui Kan, Xinying Li, Nuannuan Chen.

**Formal analysis:** Shaokui Kan, Xinying Li, Nuannuan Chen, Yingli Zhang.

**Funding acquisition:** Shaokui Kan.

**Investigation:** Shaokui Kan, Xinying Li, Nuannuan Chen.

**Methodology:** Shaokui Kan, Xinying Li.

**Project administration:** Shaokui Kan, Yingli Zhang.

**Resources:** Shaokui Kan, Yingli Zhang.

**Software:** Shaokui Kan.

**Supervision:** Shaokui Kan, Xinying Li, Nuannuan Chen, Yingli Zhang.

**Validation:** Shaokui Kan, Xinying Li.

**Visualization:** Shaokui Kan, Xinying Li.

**Writing – original draft:** Shaokui Kan, Xinying Li, Nuannuan Chen, Yingli Zhang.

**Writing – review & editing:** Shaokui Kan, Xinying Li, Nuannuan Chen.
